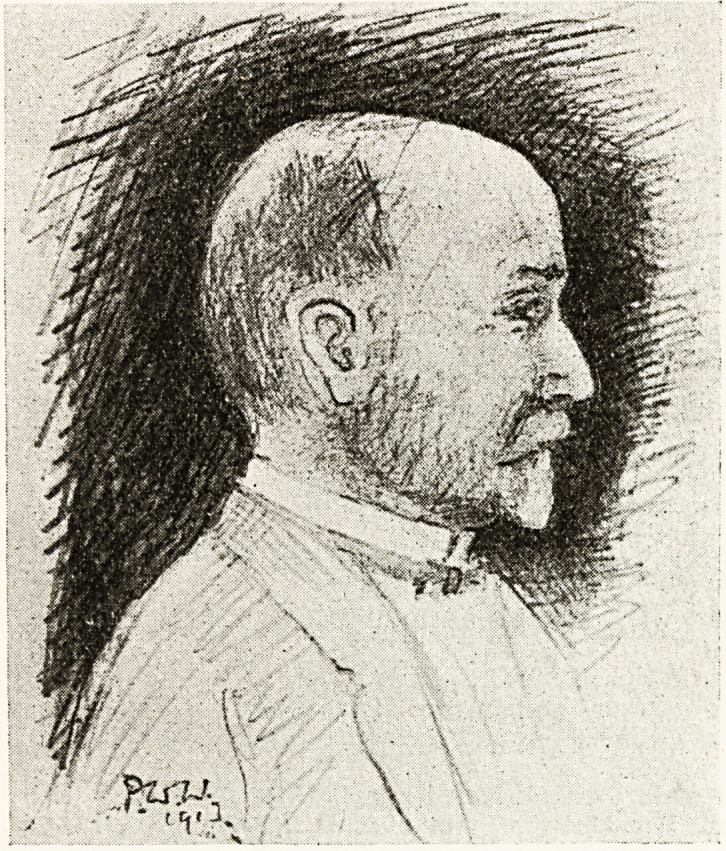# Robert Shingleton Smith

**Published:** 1922-06

**Authors:** 


### Obituary.

### ROBERT SHINGLETON SMITH, M.D., B.Sc., F.R.C.P.

Bristol has lost one of her most distinguished physicians by the death of Dr. Robert Shingleton Smith, M.D., B.Sc., F.R.C.P., which occurred on Saturday, April 15th, at his residence, "Deepholm," Clifton Park, at the age of 76. He had been in very indifferent health for the past two years owing to heart trouble, and latterly seriously ill for some months. He was a man of many parts, prominent in many spheres of life, and renowned in them all.

He was born August 24th, 1845, at Charlton Horethorn, on the Dorsetshire border of Somerset, and educated at the Taunton School, now known as Queen's College, where he gained one or more exhibitions and prizes.

While apprenticed to a doctor at Shepton Mallet, he learnt first to dispense and also the rudiments of Anatomy and Botany.

In 1862 he gained a Warneford Scholarship in King's College, London, and thus began his brilliant student career. He gained in 1863 the Junior Medical Scholarship and other prizes and distinctions. He was also working at the same time at other subjects in science. In 1864 he won the gold medal in Botany at the Apothecaries' Hall, and in 1865 the Senior Medical Scholarship in King's College.

He took the Bachelor of Science degree at the University of London with first class honours in the separate departments of Botany, Zoology, Geology and Palæontology. First class honours were also gained in Anatomy, Materia Medica and Chemistry while studying for the degree of Bachelor of Medicine—in addition to these he won prizes for essays in King's College Medical Society.

In 1867 he was appointed House Physician to King's College Hospital, and in that year he took his degree of Bachelor of Medicine in the London University, gaining the Scholarship and gold medal in Midwifery, as well as first class honours in Medicine.

In 1868 he took the M.D. of the London University, passing first and gaining the gold medal. Such an exceptionally brilliant academic career must have led up to a Physiciancy in a London hospital had he decided to wait ; but after holding a London Dispensary appointment for two years, together with the Sambrooke Registrarship in King's College, he was elected to a Resident's Post in the Bristol Royal Infirmary, and in 1873 he was appointed Honorary Physician, and thus at 28 years of age commenced practice in Clifton.

In 1874 he was appointed Lecturer in Physiology at the Medical School. It was most fortunate that he was available for this very important post. The subject was being rapidly developed by most able scientists in this country and abroad. The teaching in many of the London hospitals was passing from the members of the Medical Staff to special teachers in Physiology. Smith was quite capable of dealing with the subject up to a full medical standpoint, and until 1887 he continued to study, teach and lecture on Physiology to the satisfaction of the examining bodies and with very successful results on the part of his students. No one in Bristol at that time could have so satisfactorily undertaken these duties, and his colleagues at the Medical School and University College at that time were under a deep obligation to Shingleton Smith for undertaking this unremunerative and very strenuous work.

In 1887 he became Lecturer at University College in Pathology, and a year later was made conjoint Lecturer in Medicine with Dr. Markham Skerritt. Later they became Professors, and in 1894 Smith resigned. When the University of Bristol was founded in 1908 he was designated Emeritus Professor of Medicine. The degree of M.D. *honoris causâ* was conferred upon him by the University in 1912.

Just at the time when Smith joined the Honorary Staff at the Royal Infirmary a long-felt need in connection with the Medical School had become urgent. The lecturers of the School, feeling the inadequate accommodation in the old Park where good work had been attempted since 1833, decided to make an appeal to the public to obtain funds for a new building. A preliminary circular was drawn up, but at a meeting held on February 3rd, 1873, Mr. Thomas Coomber, Lecturer on Chemistry at the School, suggested that a School for Science should be started in Bristol, of which the Medical School should be a part. The idea was warmly taken up by the Faculty of the School; meetings were held and interest was spread among the citizens, and the idea taken up by those interested in education and by members of the older Universities.

On June 11th, 1874, a public meeting convened at the Victoria Rooms to promote the establishment of a "College of Science and Literature for the West of England and South Wales" was very largely attended.

A preliminary circular had been issued, and it was signed for the Local Committee by Gilbert Elliot, Dean of Bristol,

Chairman ; Wm. Proctor Baker, Wm. Lant Carpenter, Lewis Fry, Honorary Secretaries ; R. Shingleton Smith, Secretary. The first resolution passed at the meeting in favour of the founding of such a College was by Professor Williamson, President of the British Association, and was seconded by Professor Jowett, Master of Balliol, and supported by Mr. E. A. Freeman, Bishop Temple, W. B. Carpenter, Professor Roscoe, of Manchester, and others. Thus the proposal of Thomas Coomber, which was the outcome of an effort on the part of the lecturers of the Bristol Medical School, resulted in the foundation of University College, the forerunner of the University of Bristol. Shingleton Smith was Secretary to the organising Committee of the College. He was throughout strongly in favour of the Medical School being an integral part of the new College, and others agreed with him. Many of the Medical School lecturers, however, did not share this view, and for several years the institutions were merely affiliated and worked in some co-operation. It was a lasting disappointment that the Medical School held aloof from University College when the latter was first founded.

The first Calendar of the University College was published in 1877. The College was then located in Park Row, while the Medical School continued its work in the Old Park. Within a year or two, however, most of the teaching was done in the new University College buildings in Tyndall's Park, and actual incorporation of the two bodies (the School becoming a part of the College) took place in 1892.

A Medical and Chirurgical Society had formerly existed in Bristol in which excellent work had been done, but it had long ago ceased to exist. The first and only volume of Bristol Royal Infirmary Reports was edited 1878–1879 by Dr. Spencer and Greig Smith.

On February 20th, 1874, a printed circular letter was issued after consultation among the more scientific members of the profession, signed by Nelson Dobson, Shingleton Smith and W. H. Spencer, stating that it is proposed to establish for Bristol and Clifton a new Medical Society, the prominent feature of which will be the development of the pathological and clinical resources of the place, but formed on a basis sufficiently broad to include all the departments of scientific and practical medicine. A preliminary meeting was held, presided over by Crosby Leonard, at which the following officers were elected : President, Dr. Brittan ; Secretary, Dr. Shingleton Smith ; with a Committee, Drs. Beddoe, Martyn and Spencer, Messrs. Dobson, Gloag and Griffiths, and in March the Bristol Medico-Chirurgical Society was duly inaugurated with its laws and regulations fully drawn up.

There can be no question as to who did the main work in carrying on the Society, and there is little doubt also as to who was its *fons et origo*. The early minutes are in Smith's handwriting, and his pen has freely annotated the original printed list of the rules.

At the end of the notes of the meeting on June 23rd, 1875, Smith writes: "The next meeting would take place in September." This meeting was actually held on October 27th, 1875, and the minutes were taken by Dr. Spencer. The President regrets that no meeting was held in September, but that in consequence of ill-health Dr. Shingleton Smith had been obliged to go abroad for the winter. He had shown symptoms of phthisis, and was sent away for a long voyage.

This must have been a very grave matter for the young physician, who had just married. But fortunately not only for himself but for his colleagues he made a good recovery. The notes of the Society are taken by Dr. Spencer, who acted as Secretary in Smith's absence for another year. The familiar handwriting appears again in the minutes of the Society on January 24th, 1877, and from this date until November, 1888, without intermission, Shingleton Smith is again the writer of all the notes. For fourteen years his untiring activity and energy were devoted to the duties of Secretary, to such good purpose that the young Society was enabled to survive all its early trials, and eventually placed upon a firm and enduring foundation.

In 1888 he was elected President of the Society, with Munro Smith as Secretary. Not only had he acted as Secretary, but had made many and various contributions and had frequently taken part in debates.

Meanwhile the Bristol Medico-Chirurgical Society and its energetic Secretary had been the means of still further developments in the medical history of Bristol.

Dr. Henry Edward Fripp in his presidential address, 1878, suggested the publication of *Transactions of the Bristol Medico-Chirurgical Society*. He said, "However unpretending as a literary effort, the publication of *Transactions* by a Society marks its growing self-consciousness of present active power . . . . and might be welcomed as a proof that the medical centre of the West of England maintains its claim to be considered a teaching power, fairly abreast with the general progress of science, and able to sustain its old repute."

It was decided to carry out the President's suggestion, and papers were selected from among the proceedings during the year 1874–1878. This volume was issued in the latter year. In 1882 the second volume of the *Transactions* should have been issued, but it seemed to several of our members inexpedient to publish papers so long after they had been read, and it was thought that an improved form of publication might be issued at shorter intervals.

At a meeting held on October 11th, 1882, a resolution was proposed by Dr. Beddoe, seconded by Mr. Greig Smith, " That in the opinion of this Society the time has come when the various medical and surgical interests of the West of England ought to be united by the publication of a periodical journal which might appear under the auspices of the Medico-Chirurgical Society." The scheme was reported on and *The Bristol Medico-Chirurgical Journal* was launched, with Greig Smith as Editor and L. M. Griffiths as Publishing Secretary, and under this extremely able management the quarterly *Journal* continued to flourish for nine years. In its very first number, July, 1883, the first paper was by Shingleton Smith on " The proofs of the existence of a Phthisical Contagion." It was a masterly summary, based on all the best scientific knowledge of the day, and supported by his own microscopical investigation, with an epitome of 49 of his cases where he had found tuberculosis bacilli, and 23 cases in which he had failed to find them in cases in lung affection which had not typical symptoms of phthisis. He says the supreme question is not merely whether tuberculosis is infectious, that seems proved, but whether the bacillus of Koch is the infective agent, and he sums up, " If the observations (he has quoted) be accurate, there is only one conclusion—that these bacilli cause tuberculosis." This paper was considerably in advance of the knowledge of the day, and was not in accord with the opinions held in regard to tuberculous disease by many skilled clinical physicians at that time. The subject of his Presidential Address on October 10th, 1888, was on " Some recent developments of the Germ Theory, more particularly in relation to the treatment of Phthisis."

Nearly twenty years afterwards he was most active in assisting Dr. Lionel Weatherly when that gentleman started the campaign which led to the establishment of the Winsley Sanatorium for consumptives, which was opened in 1904, and of which Shingleton Smith became Chairman of Committee, and afterwards President. His professional experience had taught him the need of such a place, and he threw his whole heart into the movement. He addressed many meetings held in the district, which were arranged to arouse public opinion on the question, while he brought the weight of his influence to bear upon his friends who were in a position to lend a helping hand. His services were freely recognised by all who were anxious that adequate provision should be made in this locality for combating the ravages of this national scourge. To have a physician of his high reputation on their side was of material assistance, and in the Winsley Sanatorium we have a lasting memorial of one who delighted in doing good.

When Greig Smith had completed nine years of arduous and most successful work as Editor of *The Bristol Medico-Chirurgical Journal*, some relief was given him by the appointment of a small Editorial Committee in addition to his Assistant Editor, Mr. Griffiths. But he found himself unable to continue, and to the great regret of all he resigned the post. A very strong resolution of thanks was passed by the Society. The *Journal* had reached a very high standard of merit, was a convenient way of publishing local scientific work, and was a very valuable means by which books came for purposes of review and found their way into the Medical Library. The vacancy must be filled by a capable Editor. Shingleton Smith's name was at once thought of, but would it be possible for him to find the time? He was very busy in private practice, in addition to his daily work at the Infirmary, his lectureship at the Medical School, and his scientific enquiries. After consideration he decided to accept the post, with Mr. Griffiths as Assistant Editor and the small standing Editorial Committee. For twenty years he retained this very important position, and the *Journal* continued to flourish. On his retirement he was heartily thanked, and a letter was circulated containing the following: "The retirement of Dr. Shingleton Smith from the Editorship of *The Bristol Medico-Chirurgical Journal*, after so many years' invaluable work, is a fitting opportunity for the members of the Society and others to express their appreciation of his unremitting and important services whereby not only this Society, but the whole of the profession in Bristol and the neighbourhood has greatly benefited. When the Society was founded in 1874 Dr. Shingleton Smith was appointed the first Honorary Secretary, and it is largely through his energy and capable management that we have now such a fine Medical Library and so flourishing a local *Journal*." He was entertained at a largely attended dinner, and was presented with a silver salver and a painting that he had selected by Reginald Smith. He had previously, together with Mr. N. C. Dobson and Mr. L. M. Griffiths, been elected an honorary member of the Medico-Chirurgical Society.

Continuously working in the hospital wards on the clinical aspect of medical problems, he was a very accurate practical physician and diagnostician, but he added to this a widely-trained scientific mind. He was a very good pathologist and histologist, and made full use of the advances in chemical and microscopical science towards a knowledge of the causation as well as the cure of disease.

He was a lucid teacher and gave much time to the students, and he was always glad to help them, and any of his junior colleagues in whose professional progress he took much interest.

He was a very generous-hearted man. When he became busy as a consultant he still found time to attend on his ordinary patients, and not least those from whom he had little or no pecuniary recompense. The payment of a few guineas is a small matter to a man who is all day long concerned in the saving of life or of health. It is the progress of his patient that concerns him, not the pecuniary advantage he is going to reap. His kindly good nature had no ill word for anyone. His geniality and his power of talking on so many subjects made him deservedly popular, and some will remember the little dinners he gave to his colleagues from the country and others when they came up to our local medical meetings. His presence was always welcomed at International Congresses on Medicine as well as at those of the Medical Association. He was a member of the Committee of the Bristol Dispensary, Queen Victoria Convalescent Home, and the District Nurses' Society. He was deeply interested in astronomy and archæology, while his love of botany led him to become a Fellow of the Royal Horticultural Society. On all these subjects his knowledge was wide and accurate.

His labours in connection with the Mary Carpenter Memorial in 1878 is only one example of his many deeds of unselfishness and goodwill. He took the lion's share of the work for this Memorial, and received a very handsomely expressed letter of thanks from the members of her family.

Smith had sad trials in life. His wife was for many years an invalid, and his two daughters died at an early age. He also lost one son in the war and another was killed by an accident. The following letter shows with what courage and constancy he continued his work :—

*September 27th, 1921.*

*Dear Stock,*

*I am sorry I shall not be able to attend the meeting of the Med. Chi. Committee to-morrow evening. Please make my apologies. I have no breath for walking or talking, and am useless. Under these circumstances I think it will be best to remove my name and elect some more useful person.*

*With best wishes for the future of the Society,*

*I remain,*

*Yours very sincerely,*

*R. SHINGLETON SMITH.*

His was a fine unselfish life used for the good of others, and it is sad to feel that he suffered so much in his latter days. We offer our sympathy to his sister, his surviving son and his grandchildren.

F. RICHARDSON CROSS.

Another old and close friend writes :—

To general practitioners of Bristol and the surrounding counties Dr. Shingleton Smith was widely known as a Consultant Physician. For close upon forty years the writer of this and he were contemporaneous, and his services were repeatedly sought in cases presenting difficulty or requiring superior skill. Not once did he ever fail, and the assistance he gave was always of the greatest advantage both to the patient and the ordinary medical attendant. He had the gift of coming to a definite decision without hesitation—that is as far as obscure medical diseases can possibly permit ; but he brought his experience and quick perception to give, in nearly all cases, the malady a patient was suffering from a "local habitation and a name." Most practically, and particularly too for the relief of the sufferer, came the line of treatment to be pursued. This was invariably of the most rational nature. No long flowery prescription of former ages, but some definite remedy whose influence he supported by the theory of its therapeutic action and its effect as confirmed by his own experience of its use.

A constant association of Dr. Shingleton Smith with the writer of this in time crystallised into a long and perpetual friendship, never separated and never marred. On several occasions holidays were taken together—the Annual Meetings of the British Medical Association, with also fields of interest outside the province of medicine. One outing was a week spent in Dickens' land from Rochester to Broadstairs, visiting all the interesting places in that quarter that are mentioned in Dickens' works. What a wealth of general and scientific knowledge he displayed ! Another longer excursion was, after a fortnight's stay at Harrogate, the journey to Aberdeen to attend the quater-centenary of the University. Nothing escaped his observant eye. After a long day of travel, when one was wearying to see the Girdleness lighthouse, and turn round the angle of the coast which brings into view the fine panorama of the distant granite city, Dr. Shingleton Smith rose to his element in studying the conformation of the undulating fields and general rising of the surface of the earth leading to the cropping out of the Grampian Mountains. Freely he quoted from memory from the writings of Hugh Miller and Sir Charles Lyall, and when we skirted along and saw the rocky coast of Kincardine he was almost beside himself with sublimity and delight. It was assuredly a privilege to be the companion of a worshipper of nature such as he was ; it was God's work, not that of man, that his soul was set upon.

A large congregation, in which were many of his old medical colleagues, listened to the very touching tribute to his character paid by the Vicar at St. Paul's Church, Clifton, on the day of the funeral. One might support those lofty thoughts by a quotation which Dr. Shingleton Smith used himself many years ago in his Presidential Address at the Bath and Bristol Branch of the British Medical Association: "Let me not live after my flame lacks oil, to be the snuff of younger spirits whose apprehensive senses all but new things disdain."

J. M. RATTRAY.

The Editor was associated with his lost friend from his earliest student days, later became closer while clerking for Dr. Shingleton Smith at the Royal Infirmary until he was elected to the Honorary Staff. For many years his work with Shingleton Smith was not confined to the hospital, and he was entrusted with his private practice. It was a privilege and a constant postgraduate education to be working with such a clinician, but a far greater privilege to enjoy his intimate friendship. Shingleton Smith was ever ready to throw himself heart and soul into any new work that was presented, and when the writer was initiating the movement that crystallised in the Gloucester, Wilts and Somerset Association for the Prevention of Tuberculosis, with Dr. E. Long Fox as Chairman, Shingleton Smith's reputation as an authority on the subject was most valuable, and his enthusiastic support one of the prime factors of the successful outcome of the undertaking, viz. the establishment of the Winsley Sanatorium. No one who knew Shingleton Smith could fail to be very deeply impressed with the energy and breadth of mind of this great physician, and of the profound sense of duty, unselfishness and high principle that underlay his whole work and life. Mr. Cross has done no more than justice to his work, and in doing so he was compelled to traverse a large part of the developmental work of medicine in the West of England for several decades, for the simple reason that Shingleton Smith's inspiration was behind it all the time.

The small pencil drawing was made by the Editor on the card at the dinner held in honour of Dr. Shingleton Smith in 1913, and it endeavours to portray the twinkle that was so characteristic of the subject.

BIBLIOGRAPHY.

"Report on progress of Anatomy and Physiology," *Dobell's Report*, 1869, 1870.
"Scarlet Fever occurring in Dispensary Practice in 1869," *Med. Times and Gaz.*, 1870, ii. 263.
"Ether as an Anæsthetic," *Brit. M. J.*, 1873, i. 36.
"Clinical Lectures on a Case of Cerebral Tumour," *Ibid.*, 1874, i. 736.
"The Aspirator in Tapping the Chest," *Ibid.*, 1874, ii. 535.
"Case of Lympho-sarcoma," *Ibid.*, p. 638.
"Paracentesis Pericardii," *Lancet*, 1874, ii. 271.
"Notes on Long Voyages for Chest Disease," *Brit. M. J.*, 1876, ii. 360.
"Case of Cyanosis," *Med. Times and Gaz.*, 1877, ii. 114.
"Exophthalmic Goitre," *Ibid.*, 1878, i. 647.
"New Method of Section Cutting," *Lancet*, 1878, i. 605.
"Clinical Lecture on Acute Atrophy of the Liver," *Brit. M. J.*, 1878, i. 327.
(Co-Editor). "Annual Reports on Diseases of the Chest," Vols. I.—III., 1874–77.
"Epithelioma of Tongue and Lung," *Tr. Bristol M.-Chir. Soc.*, 1878, i. 91.
"Syphiloma of Heart," *Ibid.*, p. 117.
"Treatment of High Temperature of the Body" (Discussion), *Ibid.*, p. 129.
"Cysticercus Cellulosæ found in the Lateral Ventricle of the Brain," *Ibid.*, p. 68.
"Spinal Sclerosis," *Bristol Roy. Infirm. Rep.*, 1878–79, i. 44.
"Note on the Treatment of Gall-stone," *Lancet*, 1881, ii. 351.
"Codein in the Treatment of Diabetes," *Brit. M. J.*, 1882, i. 933.
"Case of Spasmodic Rhythmical Contractions of the Diaphragm, Recti and their Muscles," *Brain*, 1882, v. 400.
"Two Cases of Locomotor Ataxia," *Brit. M. J.*, 1882, ii. 876.
"Morbid Anatomy and Pathology of Diabetes," *Ibid.*, 1883, i. 657.
"Tubercle Bacilli in the Urine," *Lancet*, 1883, i. 942.
"The Proofs of the Existence of a Phthisical Contagion," *Bristol M.-Chir. J.*, 1883, i. 1.
"Iodoform in the Treatment of Phthisis," *Ibid.*, p. 218.
"Kairin in Typhoid Fever," *Brit. M. J.*, 1884, i. 313.
"Case of Acute Biliary Cirrhosis," *Ibid.*, p. 101.

"Diphtheria treated by Tracheotomy, Peptonised Enemata and Iodoform," *Bristol M.-Chir. J.*, 1884, ii. 61.
"Case of Sub-acute Poliomyelitis Anterior with commencing Phthisis," *Ibid.*, p. 174.
"Intestinal Obstruction" (Discussion), *Ibid.*, 1885, iii. 20.
"Cases illustrating the Action of Intra-pulmonary Injections," *Ibid.*, p. 164.
"On Intra-pulmonary Injections," *Brit. M. J.*, 1885, ii. 817.
"Aneurism of the Aorta treated by Galvano-puncture," *Bristol M.-Chir. J.*, 1886, iv. 233.
"Some recent additions to the Dietary of the Sick," *Ibid.*, p. 55.
"Iodoform in the Treatment of Tubercular Phthisis," *C. R. Cong. internat. d. Sc. méd.* (Copenh.), 1886, ii. 46.
"Treatment of Phthisis by Intra-pulmonary Injections," *Tr. Internat. M. Congr.* (Washington), 1887, i. 195.
"Cases of Phthisis treated by Carbolate of Camphor," *Bristol M.-Chir. J.*, 1888, vi. 185.
"Presidential Address to the Bristol Medico-Chirurgical Society on 'Some Recent Developments of the Germ Theory,'" *Ibid.*, p. 225.
"Some Recent Developments of the Doctrine of Contagium Vivum," *J. Micr. and Nat. Sc.*, 1890, iii. 30.
"Curious Case of Congenital Deformity," *Lancet*, 1890, ii. 817.
"Recent Work on Phthisis," *Med. Annual*, 1890–92.
"Recent Work on Pulmonary Disease," *Ibid.*, 1893.
"Antipyretics and Internal Antiseptics in Typhoid Fever," *Clin. J.*, 1893, ii. 257.
"Presidential Address to the Bath and Bristol Branch of the B.M.A. on 'Twenty-five Years of Clinical Work," 1893. (Bristol: Arrowsmith.)
"Membranous Exudations," *Clin. J.*, 1894, iv. 297.
"Stenosis and Endo-arteritis of the Pulmonary Artery," *Med. Press and Circ.*, 1894, lviii. 203; also *Brit. M. J.*, 1894, ii. 1099.
"Antiseptics in the Treatment of Phthisis." *Med. Annual*, 1894.
"The Tabetic Foot," *Clin. Sketches*, 1895, ii. 163.
"Clinical Lecture on Cardiac Disease," *Clin. J.*, 1896, vii. 141.
"Case of Double Spasmodic Torticollis," *Bristol M.-Chir. J.*, 1896, xiv. 297.
"Progress in Pulmonary Diseases," *Med. Annual*, 1896, 1897.
"The Epidemic of Typhoid at Clifton," *Brit. M. J.*, 1897, ii. 1875.
"Scurvy as a Cause of Hæmaturia," *Bristol M.-Chir. J.*, 1903, xxi. 320.
"Notes on Examination for Life Assurance," *Ibid.*, 1905, xxiii. 11; also *Med. Press and Circ.*, 1907, lxxxiii. 36; *Med. Exam. and Pract.*, N.Y., 1908, xviii. 149.
"The Long Fox Lecture on 'The Pathology and Treatment of Graves's Disease,'" *Bristol M.-Chir. J.*, 1905, xxiii. 317.

---

## Figures and Tables

**Figure f1:**